# Non-femoral focused transaxillary access in TAVI: GARY data analysis and future trends

**DOI:** 10.1007/s00392-024-02402-9

**Published:** 2024-03-04

**Authors:** Max M. Meertens, Matti Adam, Andreas Beckmann, Stephan Ensminger, Christian Frerker, Moritz Seiffert, Jan-Malte Sinning, Raffi Bekeredjian, Thomas Walther, Friedhelm Beyersdorf, Helge Möllmann, Ümniye Balaban, Kaveh Eghbalzadeh, Tanja K. Rudolph, Sabine Bleiziffer

**Affiliations:** 1 Department III of Internal Medicine, University Hospital of Cologne, Cologne, Germany; 2 Department of Cardiac and Pediatric Cardiac Surgery, Evanglish Clinical Center Niederrhein, Heart Center Duisburg, Duisburg, Germany; 3 Department of Cardiac and Thoracic Vascular Surgery, University Heart Center Lübeck, University Hospital of Schleswig Holstein, Lübeck, Germany; 4 German Center for Cardiovascular Research (DZHK), Partner Sie Hamburg-Kiel-Lübeck, Berlin, Germany; 5 Department of Cardiology, Angiology and Intensive Care Medicine, University Heart Center Lübeck, University Hospital Schleswig-Holstein, Lübeck, Germany; 6 University Heart and Vascular Center Hamburg, Hamburg, Germany; 7 Department of Cardiology, St Vinzenz Hospital, Cologne, Germany; 8 Department of Cardiology and Angiology, Robert-Bosch-Krankenhaus, Stuttgart, Germany; 9 Department of Cardiovascular Surgery, University Hospital Frankfurt and Goethe University Frankfurt, Frankfurt a. M., Germany; 10 Department of Cardiovascular Surgery, Heart Centre Freiburg University, Freiburg, Germany; 11 The Department of Internal Medicine, St.-Johannes-Hospital Dortmund, Dortmund, Germany; 12 Institute of Biostatistics and Mathematical Modelling, Goethe-University, Frankfurt, Frankfurt a. M., Germany; 13 Department of Cardiothoracic Surgery, University Hospital Cologne, Cologne, Germany; 14 Department for General and Interventional Cardiology/Angiology, Heart and Diabetes Center North Rhine-Westphalia Bochum, University Hospital of the Ruhr University, Bad Oeynhausen, Germany; 15 Department of Thoracic and Cardiovascular Surgery, Heart and Diabetes Center North Rhine-Westphalia, University Hospital Ruhr-University Bochum, Bad Oeynhausen, Germany

**Keywords:** TAVI, Transaxillary, Transapical, Transfemoral, Transaortic, Transsubclavian, GARY

## Abstract

**Background:**

In patients not suitable for transfemoral transcatheter aortic valve implantation (TAVI), several access strategies can be chosen.

**Aim:**

To evaluate the use and patient outcomes of transaxillary (TAx), transapical (TA), and transaortic (TAo) as alternative access for TAVI in Germany; to further evaluate surgical cutdown vs. percutaneous TAx access.

**Methods:**

All patients entered the German Aortic Valve Registry (GARY) between 2011 and 2019 who underwent non-transfemoral TAVI were included in this analysis. Patients with TA, TAo, or TAx TAVI were compared using a weighted propensity score model. Furthermore, a subgroup analysis was performed for TAx regarding the percutaneous or surgical cutdown approach.

**Results:**

Overall, 9686 patients received a non-transfemoral access. A total of 8918 patients (92.1%) underwent TA, 398 (4.1%) TAo, and 370 (3.8%) TAx approaches. Within the TAx subgroup, 141 patients (38.1%) received subclavian cutdown, while 200 (54.1%) underwent a percutaneous approach. The TA patients had a significantly lower 30-day survival than TAx patients (TA 90.92% vs. TAx 95.59%, *p* = 0.006; TAo 92.22% vs. TAx 95.59%, *p* = 0.102). Comparing percutaneous and cutdown TAx approaches, no significant differences were seen. However, more vascular complications occurred (TA 1.8%, TAo 2.4%, TAx 12.2%; *p* < .001), and the hospital length of stay was shorter (TA 12.9 days, TAo 14.1 days, TAx 12 days; *p* < .001) after TAx access.

**Conclusion:**

It may be reasonable to consider TAx access first in patients not suitable for TF-TAVI, because the 30-day survival was higher compared with TA access and the 1-year survival was higher compared with TAo access. It remains important for the heart teams to offer alternative access modalities for patients not amenable to the standard TF-TAVI approaches.

**Graphical Abstract:**

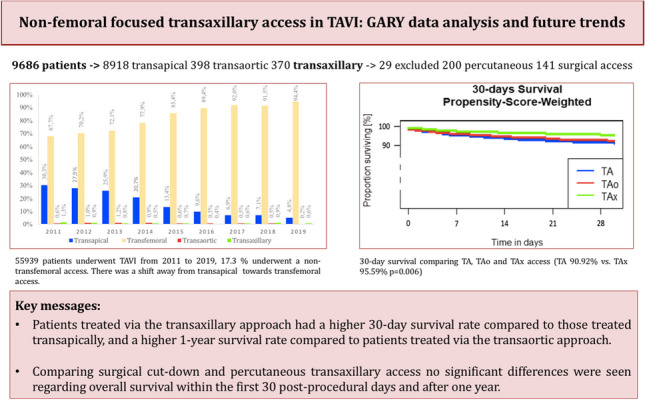

## Introduction

As transcatheter aortic valve implantation (TAVI) continues to expand to a broader and lower risk patient population and operator experiences increase constantly, the use of the transfemoral (TF) access is becoming even more dominant [1, 2]. The transfemoral access is the most chosen primary access as it is secure and the available catheter equipment is developed for this particular access strategy. This is also facilitated by data indicating that transapical (TA) and transaortic (TAo) approaches might be related to an increased risk of access complications, 30-day mortality, and stroke compared to the transfemoral (TF) approach [3, 4]. In the recently published guidelines only, the transfemoral access has a class I indication for TAVI. Non-transfemoral TAVI is only indicated (class IIb) in patients who are inoperable for SAVR and unsuitable for transfemoral access [5]. Alternative access routes, such as transaxillary (TAx), transcaval, or transcarotid access, are of growing interest for patients with relevant iliofemoral atherosclerotic disease, small iliofemoral diameters, or tortuous vessels in whom TF access is not feasible [6, 7]. The TAx access route offers an access vessel that has a diameter usually greater than 6 mm, a low calcification profile, and a short distance to the heart and is located superficially, giving the vessel a similar profile to the femoral artery [8] and the option for a procedure without general anesthesia. Comparing TAx and TF access in high-risk patients, the results regarding 30-day mortality and vascular complications were comparable [9].

Within this analysis, we aimed to investigate the adoption and outcomes with non-TF TAVI access in Germany, focusing on the axillary access route.

## Methods

### German Aortic Valve Registry (GARY)

Data of all patients who underwent non-transfemoral TAVI between 2011 and 2019 were extracted from the database of GARY which is a nationwide multicenter all-comers registry. The registry design has been previously published [10].

### Patients

We performed two separate analyses. In the first analysis, all GARY patients with TA, TAo, or TAx primary access for TAVI treated between 2011 and 2019 were included. Patients who underwent more than one intervention, with unknown access or with missing values to perform propensity score weighted analysis, were excluded. The second analysis included only the TAx patients. Two groups were generated dividing the TAx population in percutaneously and through surgical cutdown treated patients.

### Outcomes

For both analyses, we chose the same primary and secondary outcomes. The primary outcomes were 30-day and 1-year mortality. Cerebrovascular events, myocardial infarction, bleeding, vascular complications, aortic valve incompetence, and hospital length of stay were the applied secondary outcomes.

### Statistics

Continuous variables were presented as mean ± standard error, and categorical variables were described as frequencies and percentages. Two groups’ comparison for continuous variables was done by *t*-test; for more than two groups, we used an ANOVA to compare the groups. Categorical data were compared with a chi-square test.

Since the patients were not randomly assigned to the different access strategies, a weighted propensity score model was used to prevent potential bias in the comparison of patient groups induced by confounders. Patients with TA and TAo TAVI are weighted to match the characteristics of the TAx TAVI population, respectively, for comparing these three groups, and patients treated percutaneously are weighted to match the characteristics of the patients receiving surgical cutdown in the second analysis part. Possible confounders included in each propensity score model were age, sex, body mass index, peripheral arterial disease, left ventricular ejection fraction, coronary artery disease, heart rhythm at presentation, lung disease, neurological disease, diabetes, ASA, NYHA, and chronic dialysis. Differences with a two-sided* p*-value of ≤ 0.05 were considered statistically significant.

Statistical analysis was performed with SAS statistical software (version 9.4, SAS Institute, Cary, NC, USA) and R (R Foundation for Statistical Computing, Vienna Austria). The R packages “twang” and “survey” were used for calculating propensity score weights and the corresponding weighted analysis.

## Results

### Patient selection and characteristics

**Fig. 1:**
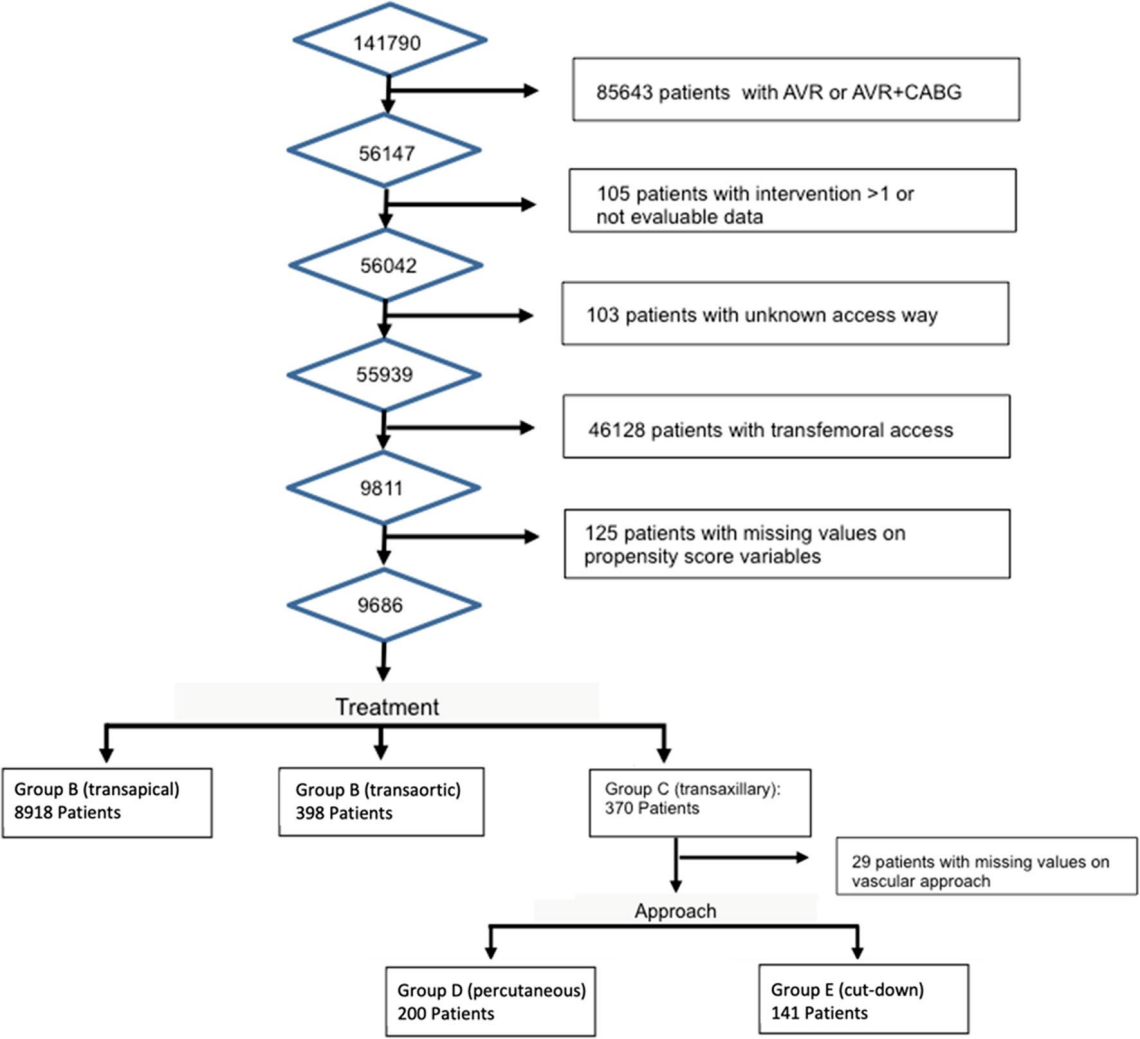
Patient selection

**Fig. 2:**
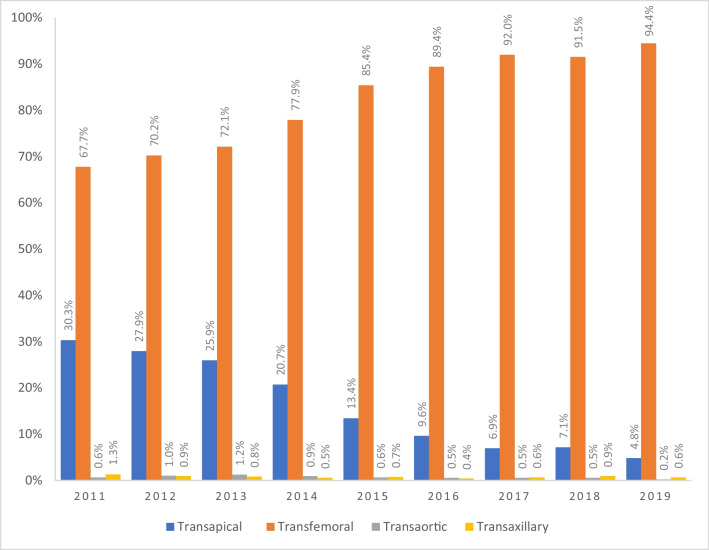
Access site usage per year

Overall, 55,939 patients underwent TAVI from 2011 to 2019, and 9686 patients (17.3%) were enrolled with a non-transfemoral access. A total of 8918 patients (92.1%) received TA, 398 (4.1%) TAo, and 370 (3.8%) TAx approaches (Fig. 1). There was a shift away from TA towards TF access over time, while TAx undulated between approximately 1.3 and 0.4% (Fig. 2). Zooming in on TAx, 141 patients underwent a surgical cutdown, 200 were treated percutaneously, and 29 patients were excluded, because their approach was unknown. The usage of non-TF access strategies declined from 33.3% in 2011 to 5.6% in 2019 (Fig. 2). Patient characteristics of all groups are presented and compared in Table 1.

**Table 1:** Patient characteristics

	TA	TAo	TAx	*p* value	Perc	Cutdown TAx	*p* value
*N*	8918	398	370		200	141	
Age (years)	79.97	79.82	79.73	0.23	79.6	79.8	.818
Female gender	39.1	39.7	38.9	0.99	38.1	37.6	.942
BMI (kg/m^2^)	26.8	26.3	26.9	< 0.001	26.8	28	.103
NYHA							
I	1.4	1.0	1.4	0.98	0.5	2.1	.421
II	4.7	5.4	4.1	0.96	3.9	2.1	.474
III	74.6	72.9	73.8	0.96	73.9	71.6	.736
IV	19.4	20.6	20.8	0.97	21.8	24.1	.711
Atrial fibrillation	28.6	27.8	27.8	0.99	77	78	.870
Hypertension	90.3	90.8	76.8	< .001	92.1	62.4	< .001
Lung disease	39.8	38.5	41.6	0.87	43.7	55.4	.121
Neurological Dysfunction	15.9	18	15.9	0.89	16	14.9	.834
Diabetes mellitus	38.8	35.4	39.7	0.71	65.2	59.6	.442
PH	18.6	20.1	24.9	0.21	21.9	25.2	.611
PAD	60.4	57.8	61.9	0.77	65.2	59.6	.621
Dialysis							
Acute	0.5	0.5	0.5	0.99	0.7	0.7	.996
Chronic	3.6	3.9	3.8	0.99	4.2	2.8	.621
EF							
≤ 30%	13.9	16.8	16.5	0.76	12.8	14.2	.787
31–50%	36.8	34.8	35.2	0.93	34	33.6	.956
≥ 51%	50.7	48.4	48.3	0.93	53.3	52.2	.893
Coronary artery disease	34	33.9	35.9	0.93	65.5	65.2	.859
Cardiogenic shock							
Yes, < 48 h	2.8	4.1	6	0.14	3.2	6.4	.359
Yes, < 21 days	14.7	11.2	13.2	0.06	18.5	26.2	.228
Yes, > 21 days	15.6	22.8	15.4	0.06	6.7	23.4	.006
ASA classification							
Class 1	0.2	0.3	0.0	0.66	0	0	1
Class 2	1.2	1.1	1.1	0.97	1.8	0	.996
Class 3	68.0	69.7	66.5	0.80	71	64.5	.360
Class 4	29.3	26.7	30	0.70	26.4	32.6	.368
Class 5	1.3	2.1	2.4	0.70	0.8	2.8	.385
Euro-score mean ± sd	28.34 ± 0.34	28.12 ± 0.37	29.10 ± 0.32	0.09	27.39 ± 1.94	29.43 ± 1.68	0.38

Values are presented in percentages unless indicated otherwise. *ASA*, American Society of Anesthesiologists; *BMI*, body mass index; *EF*, ejection fraction; *PAD*, peripheral arterial disease; *perc*, percutaneous; *PH*, pulmonary hypertension; *sd*, standard deviation; *TAo*, Transaortic; *TApi*, transapical; *TAx*, transaxillary

### Procedural characteristics and outcomes

Comparing the three access locations, the procedure time (TA 90.9 min, TAo 124.3 min, TAx 118.6 min; *p* < 0.001), dose area product (TA 4662, TAo 10969, TAx 9470; *p* < 0.001), and contrast volume (TA 104.6 ml, TAo 133.8 ml, TAx 144.8 ml; *p* < 0.001) were significantly lower in TA TAVI procedures. Also, the type of implanted prostheses differed among the access locations. TAx patients more often received self-expandable prostheses (TA 28%, TAo 49.5%, TAx 69.5%; *p* < 0.001). More intra-procedural vascular complications occurred in TAx procedures (TA 0.6%, TAo 1.4%, TAx 3.2%; *p* = 0.048).

Comparing percutaneous and surgical cutdown strategies for TAx, percutaneous procedures required significantly more contrast volume (percutaneous 165.4 ml vs. cutdown 127.9 ml; *p* < 0.001). Self-expandable aortic valve prostheses were implanted more often in cutdown TAx TAVI (percutaneous 58.2% vs. cutdown 83%; *p* = 0.002). The procedural characteristics are presented in Table 2.

**Table 2:** Procedural characteristics and outcomes

	TA	TAo	TAx	*p* value	Perc	Cutdown TAx	*p* value
*N*	8918	398	370		200	141	
Elective	83.5	81	77.3	0.21	86.1	63.8	.001
Urgent	16.5	19	22.7	0.21	13.9	36.2	.001
Procedure time (mean in minutes)	90.9	124.3	118.6	< .001	115.1	118.3	.655
Dose area product	4662	10,969	9470	< .001	8465	10,642	.271
Contrast volume (mean, sd in ml)	103.6	133.8	144.8	< .001	165.4	127.9	< .001
Valve type							
Balloon expandable	65.6	44.8	19.2	< .001	25.1	13.5	.043
Self-expandable	28.0	49.5	69.5	< .001	58.2	83	.002
Rest	6.3	5.7	11.4	.035	16.8	3.5	.003
Pericardial tamponade	0.2	1.0	0.8	.59	0	1.4	.995
Vascular complications	0.6	1.4	3.2	.048	4.7	3.5	.683

Values are presented in percentages unless indicated otherwise

*ml* mililiter, *sd* standard diviation

### Post-procedural outcomes

**Fig. 3:**
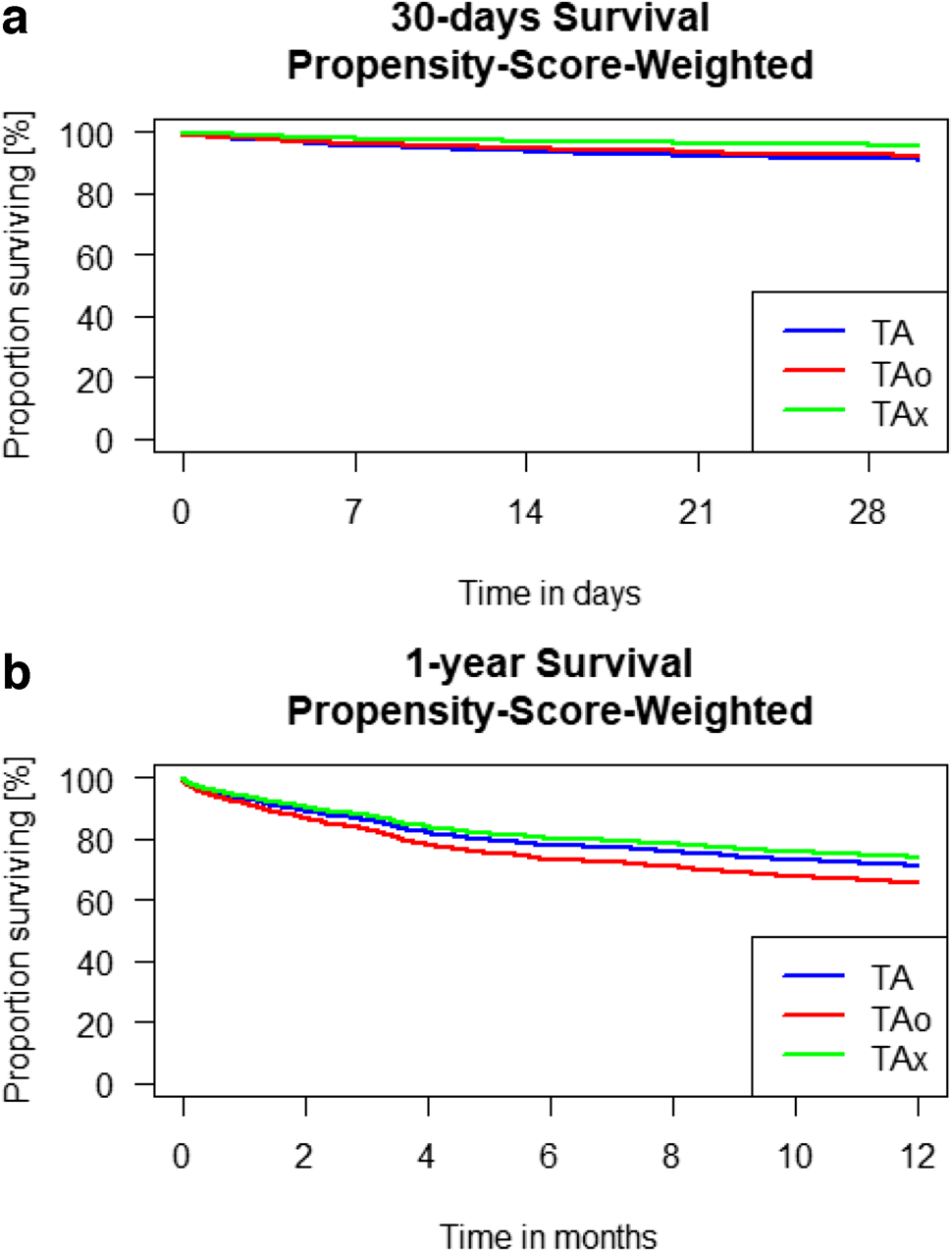
**a** 30-day survival comparing TA, TAo, and TAx access (TA 90.92% vs. TAx 95.59%, *p* = 0.006; TAo 92.22% vs. TAx 95.59%, *p* = 0.102; *p*-values from Cox-regression with propensity score weights). **b** 1-year survival comparing TA, TAo, and TAx access (TA 70.90% vs. TAx 73.70%, *p* = 0.319; TAo 65.18 vs. TAx 73.70, *p* = 0.046; *p*-values from Cox-regression with propensity score weights)

Patients undergoing TA access had worse 30-day survival than patients undergoing TAx access (TA 90.92% vs. TAx 95.59%, *p* = 0.006; TAo 92.22% vs. TAx 95.59%, *p* = 0.102; Fig. 3a). Regarding the 1-year survival, patients receiving a TAo had a worse outcome compared to TAx (TA 70.90% vs. TAx 73.70%, *p* = 0.319; TAo 65.18 vs. TAx 73.70, *p* = 0.046; Fig. 3b). More pacemaker implantations (TA 9.9%, TAo 15.3%, TAx 22.1%; *p* < 0.001) and vascular complications (TA 1.8%, TAo 2.4%, TAx 12.2%; *p* < 0.001) were reported after TAx procedures. Nevertheless, patients needed transfusion of at least 2 red blood cell products less often after TAx access (TA 30.7%, TAo 44.4%, TAx 26.8%; *p* < 0.001), and their ICU stay (TA 4.4 days, TAo 5 days, TAx 3.5 days; *p* < 0.001) and hospital length of stay (TA 12.9 days, TAo 14.1 days, TAx 12 days; *p* < 0.001) were shorter.

**Fig. 4:**
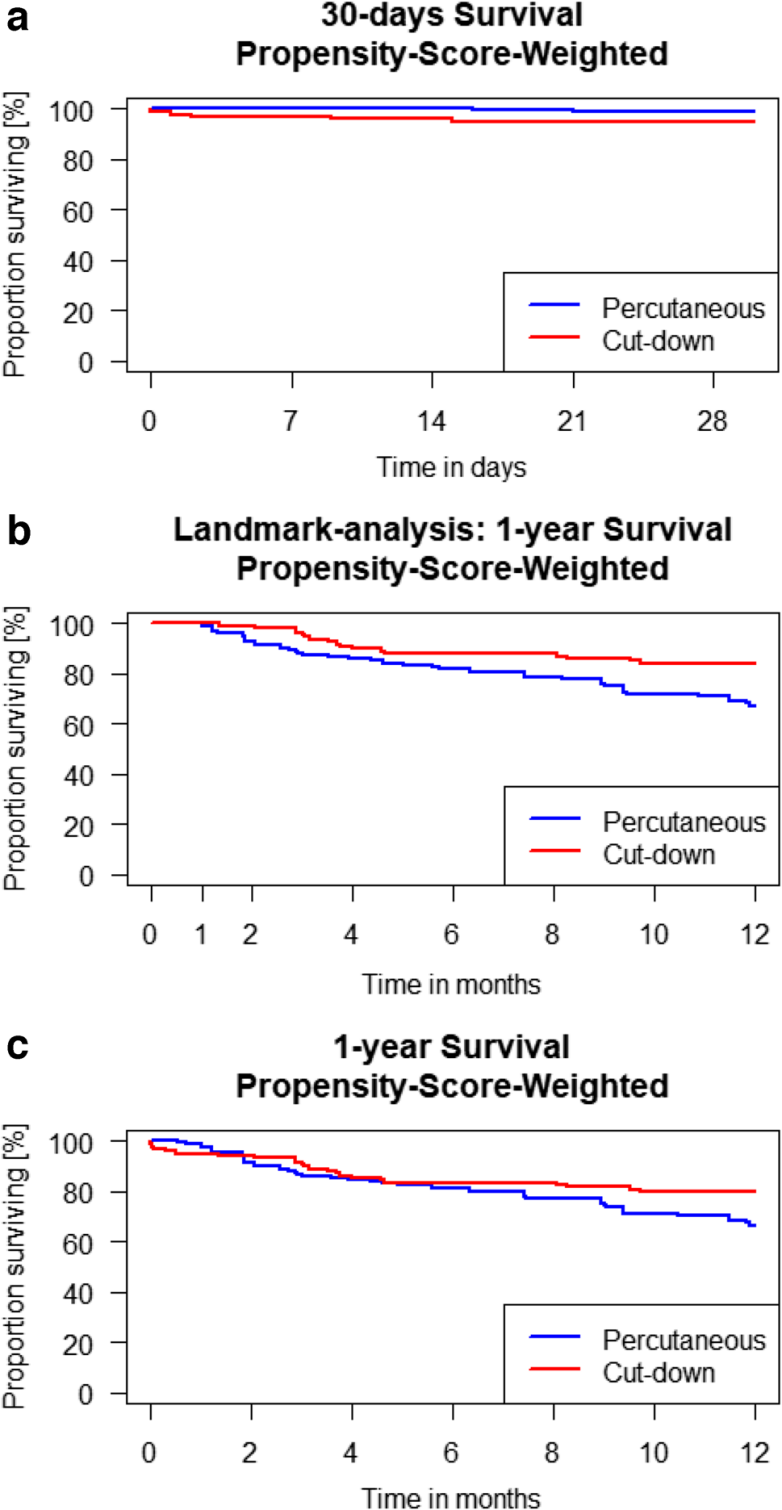
**A** 30-day survival comparing percutaneous and surgical transaxillary access (percutaneous 98.7% (CI 97.6; 99.8) vs. cutdown 94.9% (91.4; 98.7); *p* 0.057). **B** Landmark analysis for survival between 30 days and 1 year comparing percutaneous and surgical transaxillary access (percutaneous 67.1% (CI 57.0; 79.0) vs. surgical 84.0% (CI 77.3; 91.3); *p* 0.013). **C** 1-year survival comparing percutaneous and surgical transaxillary access (percutaneous 66.2% (CI 56.2; 78.0) vs. 79.7% (CI 72.7; 87.4); *p* 0.092)

Comparing post-procedural outcomes of percutaneous and surgical cutdown TAx patients, no significant differences were seen regarding the 1-year survival (percutaneous 66.2% vs. cutdown 79.9%; *p* = 0.092; Fig. 4a). Within the first 30 post-procedural days, the surgical cutdown showed non-significantly decreased survival (percutaneous 98.7% vs. cutdown 94.9%; *p* = 0.057; Fig. 4b). A landmark analysis showed that patients with surgical cutdown compared to the ones with percutaneous approach had a higher survival rate between the first 30 days and 1 year (percutaneous 67.1% vs. cutdown 84%; *p* = 0.013; Fig. 4c). A total of 16.9% of the patients receiving percutaneous access and 7.8% of the patients receiving surgical cutdown suffered a vascular complication, which did not reach statistical significance (*p* = 0.054). Regarding the other secondary outcomes, also no significant differences were observed. Table 3 presents the post-procedural outcomes.

**Table 3:** Postprocedural outcomes

	TA	TAo	TAx	*p* value	Perc	Cutdown TAx	*p* value
Stroke	1.6	0.8	3.3	0.14	6.1	1.4	.093
TIA	0.6	1.9	1.4	0.48	2.0	0	.996
Myocardial infarction	0.4	0.0	1.4	0.27	1.2	2.7	.590
New-onset Afib	5.7	7.7	5.3	0.68	6.7	3.7	.355
Pacemaker implantation	9.9	15.4	22.1	< .001	10.3	29	.164
Bleeding complication ≥ 2 RBC units	30.7	44.4	26.8	< .001	23.6	27.1	.592
Vascular complication	1.8	2.4	12.2	< .001	16.9	7.8	.054
New-onset chronic dialysis	3.1	2.4	3.3	.75	3.8	2.3	.575
Post-OP ICU stay (days)	4.4	5	3.5	< .001	3.94	3.13	.144
Post-op hospitalization (days)	12.9	14.1	12	< .001	12.5	10.8	.062
Re-hospitalization due to related complications	10.6	15.2	4.3	.22	2.9	6.7	.915
Aortic insufficiency (≥ II°)	1.6	2.8	2.5	.8	2.1	3.5	.635

Values are presented in percentages unless indicated otherwise. *Afib*, atrial fibrillation; *OP*, operation; *RBC*, red blood cell; *TIA*, transient ischemic attack

## Discussion

Although TF access is the recommended approach for TAVI, recently 6–8% of procedures in Germany were performed via a non-TF access due to anatomical peculiarities impeding TF access [5, 11]. Several factors, e.g., expansion of the TAVI indication to lower risk patients, improved deliverability of devices, up-front debulking to facilitate TF access even in hostile vascular situations, and better survival, have led to an increase of TF-amenable patients in recent years [12, 13]. In contrast to the FRANCE and STS/ACC TVT registries, in which the TAx access has gained popularity, it remained to be chosen rarely in GARY [1, 14]. Since the TA access has always been the most used alternative access strategy in Germany, it is not surprising that it remains to be the most chosen non-TF access strategy in our collected data. Both the TA and TAo access require a mini-sternotomy or a mini-thoracotomy and need to be performed under general anesthesia [11, 15]. For this reason, TA and TAo have been considered to be a more invasive procedure leading to increased complication and mortality rates [16, 17]. These fundamental disadvantages of thoracotomy and general anesthesia are not present in the third access strategy evaluated, the TAx access, which can be considered less invasive and thus potentially less harmful. Because we found a better 30-day survival compared to TA and better 1-year survival compared to TAo, because the TA/TAo patients required multiple blood products more often and the ICU and length of hospital stay were shorter after TAx access despite an increase in vascular complications, we would advocate the TAx access as the first alternative access site. However, because expertise is of particular importance, it may be useful for each heart team to gain experience with only one of the non-TF access strategies to safely treat patients who are not suitable for the standard TF-TAVI approaches.

In a subgroup analysis of a meta-analysis comparing randomized TAVI vs. Surgical Aortic Valve Replacement (SAVR) trials, the comparison of all-cause mortality between 30 days and 5 years revealed a lower incidence in patients treated via the TF approach compared to non-TF TAVI patients [18]. Siemieniuk et al. published a meta-analysis comparing TA patients to SAVR patients, which found that TA TAVI was associated with higher mortality and stroke rate [19]. To the best of our knowledge, there is currently no particular comparing the outcomes of TAVI patients with non-TF access routes other than the TA approach to those of SAVR patients. A prior analysis of TAVI patients, encompassing data from the GARY registry for the years 2011 and 2012, revealed that the chosen access route demonstrated no discernible influence on patient mortality [20]. A further analysis of the GARY registry published in the *European Heart Journal* comparing low-risk TAVI and SAVR patients showed a similar 1-year survival and higher in-hospital survival for TAVI patients [21]. Within this analysis, only a fraction of the TAVI patients were treated via non-TF access route and the authors state that non-TF access routes will have no impact on data analysis.

As a matter of concern, in previous studies, the TAx access was associated with a higher incidence of stroke. Looking into detail, the stroke incidence described in registries is just slightly above 2%, but Chung et al. described a stroke incidence of above 6.5% in 4219 TAx patients in the STS/ACC TVT registry [22, 23]. Potential risk factors for increased cerebral injuries could be manipulation/occlusion of the ostium of the vertebral artery and manipulation of the aortic arch and of the carotid/brachiocephalic ostia, possibly leading to embolism and decreased perfusion [24]. However, GARY data did not show a significant difference of stroke rates among the three access strategies. TAx access can be gained by surgical cutdown or percutaneous puncture. In TF percutaneous approach reduces the procedure time and the length of hospital stay compared to surgical cutdown without disadvantages regarding major access complications and survival [25–27]. Within the conducted analyses, no significant differences in the clinical outcomes comparing percutaneous and surgical cutdown TAx were found, but numerically more vascular complications after percutaneous access. This aligns with the data recently published from an analysis of the STS/ACC registry. Chung et al. described an increased incidence of vascular complications after percutaneous access, but without an increase in life-threatening bleedings [22]. In our landmark analysis, surgical cutdown patients had a significantly better 1-year survival (Fig. 4b). An explanation might be that the percutaneously approached population suffered insignificantly more strokes and transient ischemic attacks, which are known to result in a decreased 1-year survival after TAVI [28, 29].

The advantage of shorter procedure time and shorter length of hospital stay, which is seen after percutaneous femoral access, did not occur in our percutaneous TAx population. The procedure time of percutaneous TAx access is often prolonged by additional safety measures, eliminating this advantage over surgical cutdown. Overall, no clinically relevant and statistically significant differences were identified. Therefore, the choice to gain the TAx access percutaneously or by cutdown should be based on the skills of the operator and the anatomical challenges of the individual patient.

### Limitations

This study has several limitations. The data presented in this study are of retrospective character. Data reported from the participating centers underly a sample validation but do not undergo further adjustment. Due to the purpose of matching, patients with insufficient data were excluded. The GARY does not provide additional procedural data and outcomes such as the access site and the occurrence of a brachial plexus lesion. Regarding the choice of an alternative access strategy, it is clear that centers might have preferred an alternative access location and a preference whether to approach the axillary artery percutaneously or by cutdown; therefore, a certain selection bias might be present.

## Conclusion

The propensity score-weighted analyses suggest that it may be reasonable to consider TAx access first in patients who are not suitable for TF-TAVI, because the 30-day survival rate was higher compared with TA access and the 1-year survival rate was higher compared with TAo access. More vascular complications occurred after TAx access without an increase in ICU a hospital length of stay. Numerically higher stroke rates after TAx access are of concern and require more investigation. It remains important for the heart teams to offer alternative access modalities for patients not amenable to the standard TF-TAVI approaches.
